# Correlation between polymorphisms in toll-like receptor genes and the activity of hepatitis B virus among treatment-naïve patients: a case-control study in a Han Chinese population

**DOI:** 10.1186/s12879-018-2943-x

**Published:** 2018-01-10

**Authors:** Yong Lin, Zheng-Xiang Gao, Xu Shen, Mei-Jun Chen, Yan-Ting Li, Shu-Lian Li, Hui-Ling Lin, Qi-Feng Zhao, Fan Liu, Jian-Jun Niu

**Affiliations:** 1 Center of Clinical Laboratory, Zhongshan Hospital, Medical College of Xiamen University, 201-209 Hubin South Road, Siming District, Xiamen City, 361004 Fujian Province People’s Republic of China; 2 Institute of Infectious Disease, Medical College of Xiamen University, Xiamen, Fujian Province China; 3 School of Public Health, Fujian Medical University, Fuzhou, Fujian Province China; 4 Xiamen Huli District Maternity and Child Care Hospital, Xiamen, Fujian Province China; 5 Fujian Provincial Key Laboratory of Chronic Liver Disease and Hepatocellular Carcinoma, Xiamen, Fujian Province China

**Keywords:** Toll-like receptors, Hepatitis activity, Immune response, Hepatitis B virus

## Abstract

**Background:**

Because of the high prevalence and absence of cure for infection, chronic hepatitis B virus (HBV) infection has been acknowledged as a pressing public health issue. Toll-like receptors (TLRs) activate the human innate immune system and the polymorphisms in TLRs may alter their function. The present study aimed to investigate the association between TLR polymorphisms and disease progression of chronic HBV infection.

**Methods:**

During the study period, 211 treatment-naïve patients with chronic HBV infection were recruited, and blood samples were collected from each individual. Matrix-assisted laser desorption/ionization time of flight mass spectrometry was employed to genotype the selected TLR polymorphisms after human genome extraction. In addition, HbsAg, TNF-α, and IL-6 levels were quantified using enzyme linked immunosorbent assay (ELISA). Statistical analyses were conducted to investigate the association between TLR polymorphisms and hepatitis activity, liver function parameters, HbsAg level, and cytokine level.

**Results:**

We did not observe any mutations in rs4986790, rs4986791, and rs5743708 among all study subjects. A logistic regression revealed that mutations in rs3804099 and rs4696480 were associated with milder hepatitis activity. Consistent with the logistic regression, improved liver function parameters and reduced level of both HbsAg and cytokines were also correlated with the mutant carriers of rs3804099 and rs4696480.

**Conclusions:**

TLR mutations were significantly associated with milder hepatitis activity among patients with chronic HBV infection. Therefore, we conclude that the activation of TLR pathways may further intensify the inflammation of hepatocytes, and leads to progression of disease.

## Background

Hepatitis B virus (HBV) is a type of DNA virus that belongs to the Hepadnaviridae family, and the genome of HBV consists of small, partially double-stranded DNA containing only 3200 bp in total length. The HBV genome has four overlapping regions and encodes four genes, namely HBsAg (S), HBcAg (C), viral polymerase (P), and the X gene which is closely associated with the development of hepatocellular carcinoma [1]. Unlike most DNA viruses, the replication of HBV involves the reverse transcription of an RNA intermediate, which is more similar to retroviruses. Approximately 1 × 10^11^ viral particles are generated in an infected patient per day. Due to the poor fidelity of the polymerase, a large number of mutations occur during a highly active infection [2]. When compared with other DNA viruses, HBV possesses a 10-fold higher mutation rate [3]. The evidence suggests that HBV mutations are associated with increased replication activity, vaccine failure, carcinogenesis, occult infection, and drug resistance caused by the selective pressure of antiviral agents. Currently, nucleotide analogues below the cytotoxic threshold are widely applied in clinical practice to treat chronic HBV as well as HIV infection, because HBV and HIV polymerases share a similar function and structure [4]. The major advantage of nucleotide analogues is that they can reduce the viral titer directly and relatively fast; however, the application of nucleotide analogues has not been able to eradicate chronic HBV infection, and prolonged use is associated with the development of drug resistance which considerably reduces efficacy [5]. HBV mutations are the major factor affecting clinical outcome among patients with chronic HBV infection.

Chronic HBV infection is a pressing public health issue because of the large number of individuals affected, in addition to the unfavorable outcomes it causes: such as liver cirrhosis, fibrosis, and hepatocellular carcinoma. Approximately 240 million people suffer from chronic HBV infection worldwide, with deaths attributed to it totaling more than 686,000 people [6]. The prevalence of chronic HBV infection varies from 0.1% to 20% globally and about half of all chronic disease cases and mortality occur in mainland China. The rate of progression to chronic HBV infection is approximately 90% for perinatally acquired infection, from 20% to 50% for infections acquired at 1 to 5 years of age, and less than 5% for adult acquired infection [7]. Young individuals without vaccine protection are thus the highest risk population for developing chronic HBV infection.

Despite the great success yielded from the immunization program, a large number of patients suffer from chronic HBV infection and as such are at increased risk of cancer. Host immunity also plays a role in the progress and outcome of HBV infection [8, 9]. Toll-like receptors (TLRs) are pattern recognition receptors that recognize pathogen- and damage-associated molecular pattern molecules and thus activate the innate immune system. Toll-like receptor-mediated inflammatory signaling pathways have been correlated with a spectrum of liver diseases, such as hepatitis, liver fibrosis, cirrhosis, alcoholic and nonalcoholic liver disease, ischemia/reperfusion injury, liver regeneration, and hepatocellular carcinoma [10, 11]. Therefore, it is imperative to study the association between genetic variants in TLRs and HBV disease progression. To understand the role of genetic variants in TLRs in HBV disease progression, we conducted a case-control study among patients with chronic HBV infection in a Han Chinese population.

## Methods

### Study subjects

In total, 211 eligible subjects who were recruited from the Department of Gastroenterology, Zhongshan Hospital, Xiamen University between Nov 2015 to Jul 2016. The chronic HBV infection status was confirmed by using commercial enzyme linked immunosorbent assay kits (Wantai BioPharm, Beijing China) and quantified by using real time fluorescence quantitative PCR. Only treatment-naïve patients were enrolled to rule out the confounding effects caused by previous anti-viral treatment, allowing us to investigate the association in the chronic HBV patients with the natural state. Patients were included if following conditions were met: aged from 20 to 79 years old; HBV DNA ≥ 10^7^ copies/ml no prior history of anti-viral treatment. The exclusion criteria were as follows: the co-infection of HCV/HIV, received anti-viral treatment and refusal. All procedures involved in the present study are conformed with the Declaration of Helsinki. The study was approved by the Ethics Committee of Zhongshan Hospital, Xiamen University after formal hearing, and written, informed consent was obtained from all enrolled subjects.

### Data collection

The information on gender, age, weight, height, marital status, smoking status, alcohol consumption, and family history of hepatitis was collected from face to face interview with subjects on the date of entry by extensively trained investigators. With the permission from subjects, information on HBV infection (date of HBsAg detection, HBV genotype), and the results of liver function parameters were obtained by reviewing medical records and recorded in the unified form. The precise definition of smoking and alcohol consumption was as follows: individuals who has smoked greater than 100 cigarettes (including hand rolled cigarettes, cigars, cigarillos etc) in their lifetime and has smoked in the last 28 days., and those who consumed one or more alcohol drinks a week for over 6 months were categorized as alcohol drinkers. Fibrosis stage, hepatitis activity, and inflammation grade were established using METAVIR system [12] by liver biopsy in all included subjects.

### Sample and genotyping assay of TLRs polymorphisms

For each enrolled subject, a venous blood sample was collected on the date of entry. Blood samples were subjected to centrifugation at 4000 rpm for 10 min to separate serum and blood cells and stored at −78 °C prior to DNA extraction. Human genome DNA was done by using Magna Pure LC 2.0 system (Roche Applied Science, Mannheim, Germany). Six polymorphisms in TLRs including rs10759932, rs3804099, rs4696480, rs4986790, rs4986791 and rs5743708 were genotyped by employing Matrix-Assisted Laser Desorption/ Ionization Time of Flight Mass Spectrometry. All procedures were conducted strictly following the protocol as described by the manufacturer (Sequenom, San Diego CA, USA). A negative water control and reference DNA were employed as quality control measures during the genotyping assay. Moreover, approximately 5% of the samples were randomly selected and repeated for genotyping as duplicated controls. The genotyping call rate was 100% in accordance with the results generated from the platform.

### Determination of HbsAg serum level

Human HbsAg ELISA kit (Wantai BioPharm, Beijing China) were used to determine the serum level of surface antigen. The sensitivity and specificity of the ELISA kit we employed for the detection of HbsAg were 100% and 99.85%, respectively. The following is the detailed procedure for HbsAg quantification: (1) Firstly mark three walls as negative control, two wells as positive control and one blank. (2) Pipette 20 μl of specimen diluent to each well except the blank. (3) Then pipette 100 μl of positive control, negative control and serum to their respective wells expect blank, cover with plate sealer and incubate at 37 °C for 1 h. (4) At the end of incubation, remove and discard the plate cover, and add 50 μL HRP-conjugate into each well except blank, and mix by tapping the plate gently. (5) Cover the plate with a new plate sealer, and incubate at 37 °C for 30 min; (6) At the end of the incubation, remove and discard the plate cover. Wash each well 5 times with diluted washing buffer. Each time allow the microwells to soak for 30–60 s. After the final washing cycle, turn down the plate onto blotting paper or clean towel and tap it to remove any remainders. (7) Add 50 μl of Chromogen A and 50 μl Chromogen B solutions into each well including the blank. Incubate the plate at 37 °C for 30 min avoiding light. The enzymatic reaction between the Chromogen solutions and the HRP-Conjugate produces blue color in positive control and HBsAg positive sample wells. (8) Using a multichannel pipette or manually, add 50 μl stop solution into each well and mix gently. Intensive yellow color develops in positive control. (9) Calibrate the plate reader with the blank well and read the absorbance at 450 nm. Calculate the Cut-off value and evaluate the results within 10 min after stopping the reaction.

### *Serum level of TNF-α* (tumor necrosis factor-α) *and IL-6* (Interleukin-6) *assay*

Human TNF-α and IL-6 quantikine ELISA kits (R&D systems, Minneapolis, MN, USA) were employed to evaluate the serum level of corresponding protein. The sensitivity of the ELISA kits we employed for the detection of TNF-α and IL-6 were 5.5 pg/ml and 0.7 pg/ml, respectively. Following is the detailed procedure for TNF-α and IL-6 evaluation: (1) Firstly pipette 50 μl of assay diluent to each well. (2) Then pipette 200 μl of standard, control or serum sample (100 μl for IL-6) to each well, cover with plate sealer and incubate at room temperature for 2 h. (3) Aspirate each well and wash, repeating the process 3 times for a total of 4 washes. (4) Add 200 μL of Conjugate to each well. Cover with a new plate sealer, and incubate at room temperature for 2 h on the shaker. (5) Aspirate and wash for 4 times. (6) Add 200 μL substrate solution to each well. Incubate at room temperature for 30 min on the benchtop, and keep from the exposure of light. (7) Add 50 μL of stop solution to each well. Read at 450 nm within 30 min. Set wavelength correction to 540 nm or 570 nm. (8) Using the software provided by manufacturer to draw a standard curve and read the protein concentrations for each sample.

### Statistical analysis

Data analysis was performed by using SPSS software version 19 (IBM, Chicago, IL, USA) unless specified. Continuous variables were presented in the form of mean and standard deviation (SD) or median. As for the categorical variables, the data was presented as frequencies. Comparison was made between carriers of different genotypes of TLR polymorphisms, and student’s t-test or Mann-Whitney U test were employed to compare the continuous variables in accordance with the Gaussian distribution. Chi-squared test was used to conduct the comparison on the categorical variables. Hardy-Weinberg equilibrium (HWE) for all studied polymorphisms was calculated using an online calculator (http://scienceprimer.com/hardy-weinberg-equilibrium-calculator). Odds ratios (ORs) and 95% confidence interval (95%CI) were estimated using unconditional logistic regression analysis to evaluate the association between TLR polymorphisms and chronic HBV infection progression. The difference was considered statistically significant when the *P* value was less than 0.05.

## Results

### Characterization of study subjects

Study subjects included 211 treatment-naïve patients with chronic HBV infection. Among those, 151 were assigned to the mild chronic hepatitis group and the remaining 60 were assigned to the moderate to severe chronic hepatitis group in accordance with the METAVIR system. We enrolled only treatment-naïve patients to eliminate potential confounding caused by antiviral treatment. Simple statistical analyses were used to compare the demographic characteristics between two groups and the results were illustrated in Table 1. The proportion of males in the moderate to severe hepatitis group was significantly higher than the proportion of males in the mild chronic hepatitis group (61.6% vs. 83.3%, *P* = 0.002). The mean BMI in the mild chronic hepatitis group was significantly lower than in the moderate to severe chronic hepatitis group. Surprisingly, the proportion with a family history of hepatitis B was significantly higher in the moderate to severe hepatitis group as compared with the mild chronic hepatitis group (37.1% and 21.7%, respectively, *P* = 0.031). However, we observed no significant difference in age, marital status, smoking status, alcohol consumption, and HBV genotype distribution between individuals in the mild chronic hepatitis group and individuals in the moderate to severe group.

**Table 1:** Comparison on the characteristics of 211 treatment naïve patients by hepatitis activity

Variables	Mild chronic hepatitis	Moderate and severe chronic hepatitis	*P* value
	Number (%)	Number (%)	
Gender			
Male	93(61.6)	50(83.3)	
Female	58(38.4)	10(16.7)	0.002^a^
Age^b^	30.70(7.09)	31.10(7.86)	0.717
BMI^b^	21.40(2.94)	22.36(3.37)	0.040^a^
Marital Status			
Single	40(26.5)	19(31.7)	
Married	108(71.5)	41(68.3)	
Divorced	3(2.0)	0(0.0)	0.513
Smoking Status			
Smokers	17(11.3)	13(21.7)	
Never-smokers	134(88.7)	47(78.3)	0.051
Alcohol consumption			
No	20(13.2)	14(23.3)	
Yes	131(86.8)	46(76.7)	0.072
Family history of hepatitis B			
No	95(62.9)	47(78.3)	
Yes	56(37.1)	13(21.7)	0.031^a^
HBV genotype			
Genotype B	123(81.5)	48(80.0)	
Genotype C	28(18.5)	12(20.0)	0.808

^a^ *P < 0.05*

^b^Data was presented in the form of mean (standard deviation)

### TLR polymorphisms and hepatitis activity

Table 2 displays the frequency of all genotyped TLR polymorphisms in mild chronic hepatitis group and the moderate to severe chronic hepatitis group. Surprisingly, we observed no mutation in rs4986790, rs4986791, and rs5743708 in the study population; therefore, only data on rs10759932, rs3804099, and rs4696480 were presented in Table 2 and in the rest of the results section. No evidence was found for a significant difference in genotype distribution of rs1075993 between the two groups, as well as in the logistic regression (*P* > 0.05). Regarding the rs3804099 variant, we observed a protective effect against hepatitis progression in both CT heterozygous and CC homozygous (OR 0.44, 95% CI: 0.22–0.89) and (OR 0.14, 95% CI: 0.05–0.43), respectively. Similarly, the favorable genotype TT homozygous at rs4696480, which significantly reduces the possibility of disease progression, also had a protective effect (OR 0.35, 95% CI: 0.15–0.80). However, no association was found for CT heterozygous by logistic regression, and the distribution between the two groups was not significantly different (*P* > 0.05).

**Table 2:** Association between TLR polymorphisms and hepatitis activity

Genotype	Mild chronic hepatitis	Moderate and severe chronic hepatitis	Odds Ratio (95%CI)
	Number (%)	Number (%)	
rs1075993			
TT	83(55.0)	25(41.7)	1(Ref.)
CT	64(42.4)	32(53.3)	1.66(0.90–3.08)
CC	4(2.6)	3(5.0)	2.49(0.52–11.88)
rs3804099			
TT	60(39.7)	41(68.3)	1(Ref.)
CT	50(33.1)	15(25.0)	0.44(0.22–0.89)^a^
CC	41(27.2)	4(6.7)	0.14(0.05–0.43)^a^
rs4696480			
CC	83(55.0)	25(41.7)	1(Ref.)
CT	64(42.4)	32(53.3)	0.98(0.48–1.98)
TT	4(2.6)	3(5.0)	0.35(0.15–0.80)^a^

^a^ *P < 0.05*

### Comparison of liver function parameters by TLR polymorphisms

**Fig. 1:**
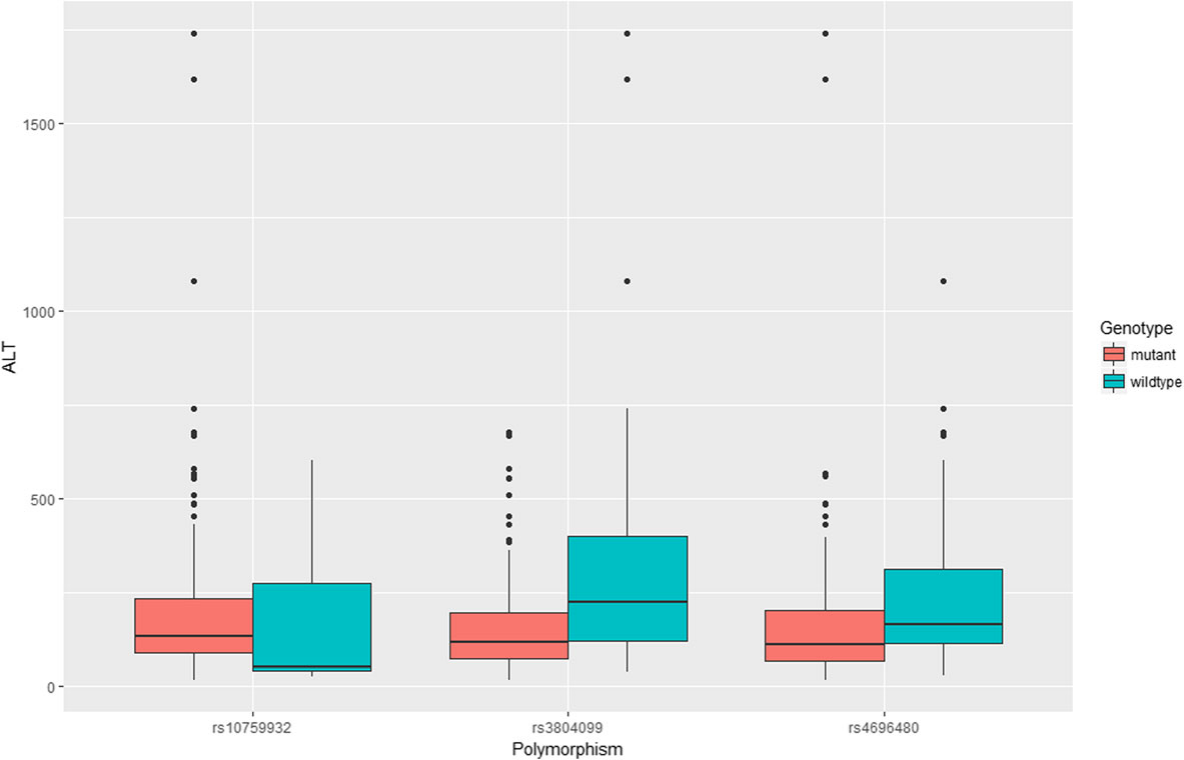
Comparison of serum levels of ALT between the carriers of wild type of TLR polymorphisms and the mutants among 211 treatment naïve patients

**Fig. 2:**
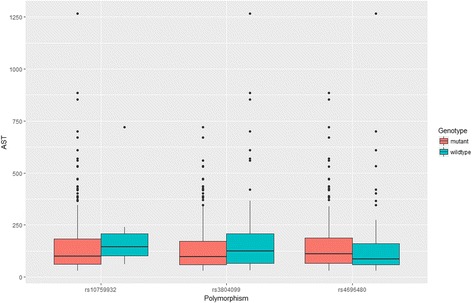
Comparison of serum levels of AST between the carriers of wild type of TLR polymorphisms and the mutants among 211 treatment naïve patients

We employed the recessive model to combine homozygous wild type and heterozygotes as one group, and the rest of the statistical analysis was based on the comparison between homozygous mutation carriers and remaining subjects. The Student’s t-test was employed to estimate the difference in liver function parameters between the wild type and mutant groups. As compared with rs10759932 mutants, wild type carriers had significantly higher globulin levels. As for the rs3804099, we observed significant elevation in both Alanine aminotransferase (ALT) and Aspartate aminotransferase (AST) among the wild type carriers when compared with their counterparts: the mean (and standard deviation) of ALT and AST level in wild type carriers were 340.91 (362.01) and 231.42 (267.27), respectively, while the corresponding figures were reduced to 156.50 (123.43) and 145.99 (131.27) in mutant carriers (See Table 3, Figs. 1 and 2). As shown in Table 4, we found a significant elevation in ALT level when comparing the subjects with different rs4696480 genotypes; however, no significant difference was observed in AST level. In addition, the rs4969480 mutant also maintained a higher level of albumin (*P*<0.05).

**Table 3:** Comparison on liver function parameters by TLR polymorphisms (rs10759932 and rs3804099) in recessive model

Variables	rs10759932 wild type	rs10759932 mutant	*P*	rs3804099 wild type	rs3804099 mutant	*P*
Total bilirubin(umol/L)	18.04(6.15)	21.87(23.24)	0.665	26.56(40.20)	20.43(15.08)	0.320
Direct bilirubin(umol/L)	3.24(2.27)	5.65(14.20)	0.655	8.45(24.88)	4.79(8.98)	0.337
Indirect bilirubin(umol/L)	14.80(5.29)	16.98(11.51)	0.618	19.98(19.24)	16.08(7.90)	0.337
ALT(U/L)	187.00(216.38)	196.13(212.91)	0.911	340.91(362.01)	156.50(123.43)	0.002^a^
AST(U/L)	220.57(228.28)	162.275(170.61)	0.380	231.42(267.27)	145.99(131.27)	0.043^a^
Total protein(g/L)	78.00(5.26)	73.79(6.45)	0.090	72.44(11.15)	74.34(4.36)	0.270
Globulin(g/L)	45.43(5.00)	41.86(4.35)	0.035^a^	41.98(7.26)	42.30(3.27)	0.996
Albumin(g/L)	32.57(3.87)	32.11(4.13)	0.772	31.27(3.50)	32.36(4.24)	0.080

^a^ *P < 0.05*

**Table 4:** Comparison on liver function parameters by TLR polymorphism rs4696480 in recessive model

Variables	rs4696480 wild type	rs4696480 mutant	*P*
Total bilirubin(umol/L)	21.29 ± 20.31	21.96 ± 24.13	0.840
Direct bilirubin(umol/L)	5.47 ± 12.62	5.62 ± 14.64	0.941
Indirect bilirubin(umol/L)	17.33 ± 12.60	16.70 ± 10.73	0.706
ALT(U/L)	244.61 ± 198.66	171.61 ± 215.66	0.018^a^
AST(U/L)	152.33 ± 190.18	170.11 ± 163.32	0.482
Total protein(g/L)	73.39 ± 4.88	74.21 ± 7.10	0.386
Globulin(g/L)	42.53 ± 3.27	41.71 ± 4.86	0.204
Albumin(g/L)	30.86 ± 4.09	32.76 ± 3.99	0.001^a^

^a^ *P < 0.05*

### Comparison of HbsAg level by TLR polymorphisms

**Fig. 3:**
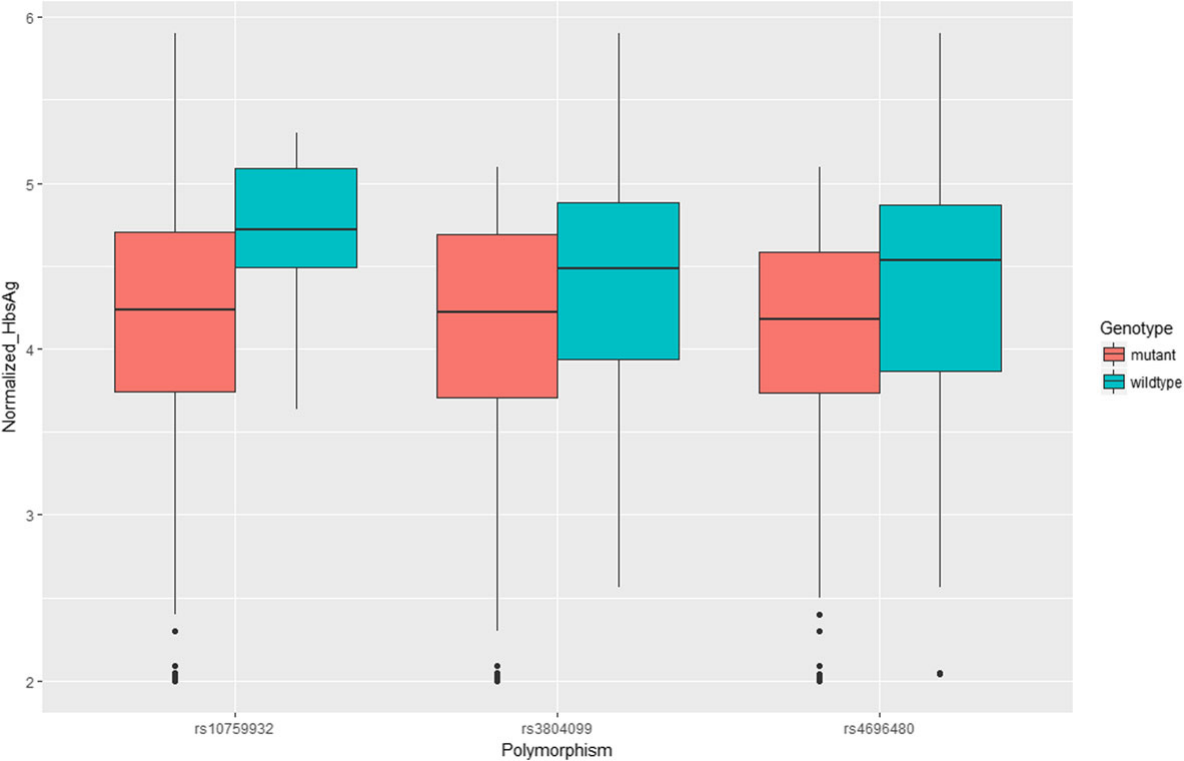
Comparison of serum levels of HbsAg between the carriers of wild type of TLR polymorphisms and the mutants among 211 treatment naïve patients

The difference in the HbsAg concentration between carriers with different genotypes was conducted by using Student’s t-test after data normalization. As shown in Table 5 and Fig. 3, mutations in rs3804099 and rs4696480 reduced serum level of HbsAg, and the results were consistent with those generated by logistic regression. With regard to rs1075993 genotypes, no significant difference was found between groups, which was similar to previous findings.

**Table 5:** Association between normalized HbsAg level and TLR polymorphisms

Genotypes	Normalized HbsAg level (ng/ml)
rs1075993 wild type	4.69(0.57)
rs1075993 mutant	4.11(0.77)
*P*	0.051
rs3804099 wild type	4.37(0.78)
rs3804099 mutant	4.07(0.76)
*P*	0.014^a^
rs4696480 wild type	4.35(0.80)
rs4696480 mutant	4.02(0.73)
*P*	0.003^a^

^a^ *P < 0.05*

### Comparison of TNF-α and IL-6 serum level by TLR2 polymorphisms

We compared TNF-α and IL-6 levels by different TLR2 genotypes. Consistent with our previous findings in HbsAg level, we did not observe a significant difference in TNF-α or IL-6 level by rs1075993 genotypes. In addition, null association between TNF-α level and rs3804099 genotypes was also found. However, rs3804099 mutation carriers had a significantly lower level of IL-6 (5.66 ± 2.68) than the carriers of rs3804099 wild type (6.38 ± 2.13) (*P* = 0.031). We observed a significant reduction in both TNF-α (2.66 ± 1.09) and IL-6 (5.36 ± 1.86) level for rs4696480 genotypes, between the mutant carrier and their counterparts (TNF-α: 3.39 ± 1.60; IL-6: 6.71 ± 2.73; *P* < 0.001). Detailed data are shown in Table 6.

**Table 6:** Comparison on TNF-α and IL-6 level by TLR polymorphisms

Genotypes	TNF- α(pg/ml)	IL-6(pg/ml)
rs1075993 wild type	2.94 ± 1.48	6.18 ± 2.47
rs1075993 mutant	3.13 ± 1.34	5.87 ± 2.39
*P*	0.330	0.357
rs3804099 wild type	3.13 ± 1.47	6.38 ± 2.13
rs3804099 mutant	2.91 ± 1.35	5.66 ± 2.68
*P*	0.262	0.031^a^
rs4696480 wild type	3.39 ± 1.60	6.71 ± 2.73
rs4696480 mutant	2.66 ± 1.09	5.36 ± 1.86
*P*	<0.001 ^a^	<0.001^a^

^a^ *P < 0.05*

## Discussion

We investigated the association between TLR polymorphisms and hepatitis activity, liver function parameters, and HbsAg level among patients with chronic HBV infection. As expected, we did not observe any mutation in rs4986790, rs4986791, and rs5743708 in the study population. These results are consistent with previous findings conducted by Hang et al. among Chinese cotton and silk textile workers over 20 years of observation [13]. These results are also consistent with the genotype distribution estimated in the Japanese population [14], suggesting the reliability and validity of the genotyping method we employed. Despite the absence of a mutation on several polymorphisms we investigated, the principle finding of this study is that both rs3804099 and rs4696480 in TLR2 mutant carriers present improved liver function parameters and reduced level of HbsAg, suggesting the mutations on TLR genes may have the potential to reduce the inflammatory damage that occurs in chronic HBV infection. The logistic regression results further demonstrate that the mutation in rs3804099 and rs4696480 were inversely associated with hepatitis activity as evaluated by METAVIR.

There has been substantial research undertaken on the role of TLRs related immune response with presence of pathogens, such as HBV. TLR2 has been correlated with the inhibition of HBV replication in human hepatoma cells [15]. However, our results indicate that both synonymous mutation and polymorphism in the promoter of TLR2 were associated with milder hepatitis activity. The concept of TLR2 activation as a protective factor has been recently challenged: Isogawa et al. [16] demonstrated that TLR2 activation was not able to restrain HBV infection in mice in vivo. Moreover, TLR2 activation leads to the production of pro-inflammatory cytokines such as IL-6 and TNF-α in hepatic non-parenchymal cells and hepatocytes [17], and it has been generally acknowledged that chronic inflammation is closely associated with cellular damage and even carcinogenesis in the presence of HBV infection. Our findings are consistent with previous studies. We found that the mutations in rs3804099 and rs4696480 were associated with a suppressed level of TNF-α and IL-6 among patients with chronic HBV infection as compared with wild type carriers. Further, no significant difference was observed in comparison of TNF-α and IL-6 level by rs1075993 genotypes, which was similar to the results of the liver function parameter comparison. In sum, our results indicate that the mutation in rs3804099 and rs4696480 leads to relieved inflammation, and the pro-inflammatory cytokines were also reduced in the TLR2 mutant carriers. Others have found evidence of increased TLR2 level associated with higher serum ALT concentration and advanced hepatitis activity in an Iranian population [18]. Consistent with the literature, improved liver function parameters were observed among those with the TLR2 mutation. Although the mutations we investigated were either synonymous mutations or in the promoter region, a possible explanation for our results is that the mutations are associated with down-regulated transcription of TLR2, and with reduced TLR2 level. Under such circumstances, the pro-inflammatory cytokines were partially inhibited. Consequently, the inflammation and damage were relieved by the TLR2 mutation.

There is a consensus that HbsAg is the most abundant protein and classic hallmark of HBV infections. The concentration of HbsAg has been correlated with disease progression, as well as with copies of HBV DNA in serum [19], and thus eradicating HbsAg has been the ultimate purpose of antiviral treatment against HBV infection. We found that TLR2 mutations were inversely associated with HbsAg level, a finding supported by Visvanathan et al. [20]. The enhanced HBV replication was associated with up-regulation of the TLR2 pathway leading to increased TNF-α production. Thus, it seems likely that such connections existing between TLR2 and hepatitis activity were mediated by the production of pro-inflammatory cytokines.

## Conclusions

We observed a positive association between TLR2 polymorphisms and hepatitis activity. With the evidence acquired from this study, it is possible to identify the patients among those with chronic HBV infection that are likely to progress to an advanced stage, in order to provide targeted interventions. The underlying mechanism between cytokines and TLR2 polymorphisms has yet to be identified. Therefore, further investigation is needed to address this question.

## Abbreviations

ALT: Aspartate transaminase
AST: Alanine transaminase
DNA: Deoxyribonucleic acid
ELISA: Enzyme-linked immunosorbent assay
HbsAg: Hepatitis B surface antigen
HBV: Hepatitis B virus
HCV: Hepatitis C virus
HIV: Human immunodeficiency virus
HWE: Hardy-Weinberg equilibrium
RNA: Ribonucleic acid
TLR: Toll-like receptors
